# Frozen by Heating: Temperature Controlled Dynamic States in Droplet Microswimmers

**DOI:** 10.1002/adma.202416813

**Published:** 2025-03-04

**Authors:** Prashanth Ramesh, Yibo Chen, Petra Räder, Svenja Morsbach, Maziyar Jalaal, Corinna C. Maass

**Affiliations:** ^1^ Physics of Fluids Group Max Planck Center for Complex Fluid Dynamics and J. M. Burgers Center for Fluid Dynamics University of Twente PO Box 217 Enschede 7500AE Netherlands; ^2^ Max Planck Institute for Dynamics and Self‐Organization Am Faßberg 17 37077 Göttingen Germany; ^3^ Max Planck Institute for Polymer Research Ackermannweg 10 55128 Mainz Germany; ^4^ University of Amsterdam Science Park 904 Amsterdam 1098 XH Netherlands

**Keywords:** active matter, microswimmers, self‐propelling droplets, temperature controlled dynamic states

## Abstract

Self‐propelling active matter relies on the conversion of energy from the undirected, nanoscopic scale to directed, macroscopic motion. One of the challenges in the design of synthetic active matter lies in the control of dynamic states, or motility gaits. Here, an experimental system of self‐propelling droplets with thermally controllable and reversible dynamic states is presented, from unsteady over meandering to persistent to arrested motion. These states are known to depend on the Péclet number of the molecular process powering the motion, which can now be tuned by using a temperature sensitive mixture of surfactants as propulsion fuel. The droplet dynamics are quantified by analyzing flow and chemical fields for the individual states, comparing them to canonical models for autophoretic particles. In the context of these models, in situ, the fundamental first broken symmetry that translates an isotropic, immotile base state to self‐propelled motility, is experimentally demonstrated.

## Graphical abstract

In the design of smart materials based on active agents or microswimmers, the gaits or motile states of the agents are to be controlled, preferably by external parameters that do not require modifying the composition of the system. A facile, universal parameter is temperature. Here, in the experimental model system of self‐propelling active emulsions, a temperature sensitive mixture of co‐surfactants is used as fuel for propulsion. It is demonstrated that three distinct states of reorienting, then straight motion, and subsequent arrest, can be accessed, counterintuitively, with increasing temperature.

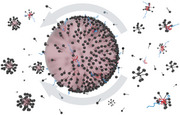

## 1 Introduction

Active matter is defined by the nonlinear conversion of free energy on the molecular scale into macroscopic dynamics:^[^ ^1^ ^]^ these nonlinear dynamics pose a challenge to the control of active agents, as small changes in system parameters can crucially tip the system over into a new dynamic equilibrium, for example from metastable inactivity to a state of directed motility. More so, like a sorcerer's apprentice^[^ ^2^ ^]^ we find that stopping this motion is not a trivial task; after all, one is required to deplete an energy reservoir or reverse a dynamic instability.^[^ ^3^ ^]^ This kind of control is important in the design and study of artificial and biological microswimmers, their theoretical modeling, experimental realization, and, ultimately, to provide design principles and dynamic control for technological application.^[^ ^4^, ^5^ ^]^

Autophoretic particles^[^ ^6^, ^7^, ^8^, ^9^ ^]^ and, particularly, their experimental counterpart, active droplets propelled by spontaneously self‐generated chemophoretic flows,^[^ ^10^, ^11^, ^12^, ^13^, ^14^, ^15^, ^16^, ^17^, ^18^, ^19^ ^]^ are popular active matter models driven by purely physicochemical mechanisms, with typical sizes ranging between ≈30μm up to several hundred μm. Generally, their dynamics are characterized by a dimensionless Péclet number *Pe* = *U*_*c*_ *R*/*D* representing the ratio of advective and diffusive transport of chemical fuel,^[^ ^20^ ^]^ with *U*_*c*_, *R* and *D* quantifying the characteristic flow speed, droplet radius and the fuel's diffusivity. With increasing *Pe*, autophoretic particles first transition from *passive* isotropic chemical conversion to *active* self‐propulsion, and further from persistent to unsteady motion via a sequence of broken symmetries and interfacial flow modes of increasing complexity.^[^ ^18^, ^20^, ^21^, ^22^, ^23^, ^24^, ^25^ ^]^

Recent studies have investigated the control of speed and dynamic states of such microswimmers in response to externally applied stimuli such as temperature^[^ ^26^, ^27^ ^]^ or illumination.^[^ ^28^, ^29^, ^30^, ^31^, ^32^, ^33^, ^34^, ^35^ ^]^ On heating, physical intuition might suggest that motion should accelerate and destabilize, either by the increase of translational and rotational diffusion with decreasing viscosity, or by increased activity from the molecular thermodynamics driving the motion. However, the nonlinear dynamics of self‐organized activity can drive counter‐intuitive effects, as we have previously found for active droplets which destabilize with increasing viscosity due to an increase in *Pe*.^[^ ^24^ ^]^

In this study, we explore another counter‐intuitive response, this time to increasing temperature. We study active droplets using a temperature sensitive combination of co‐surfactants in aqueous solution as a fuel medium. With increasing ambient temperature we find a transition between distinct dynamic states from unsteady to oscillatory to steady, straight swimming to eventual arrest, which is reversible and cyclic with temperature. Notably, by this method we are able to drive *Pe* across the critical activity threshold: we experimentally observe, live and in situ, the fundamental first symmetry breaking of the inactive isotropic base state into directed self‐propulsion, analyzing both chemical and hydrodynamic fields. This observation requires an undisturbed, unchanged ambient fuel medium, which we engineer by exploiting temperature dependent surfactant‐polymer interactions^[^ ^36^ ^]^ that were previously not considered in active droplets—opening new design possibilities to control the motion of micro‐droplets as active agents in smart materials driven by purely physicochemical mechanisms.^[^ ^37^ ^]^

## 2 Results and Discussion

### 2.1 Self‐Propelling Droplets in Co‐Surfactant Solutions

Our experimental system consists of oil droplets ((S)‐4‐Cyano‐4’‐(2‐methylbutyl)biphenyl, CB15) immersed in an aqueous solution of the ionic surfactant tetradecyltrimethylammonium bromide (TTAB) at 9–15 wt% (267–445mM) and the triblock copolymer Pluronic F127 (PF127) at 4 wt%, or 3mM.

**Figure 1:**
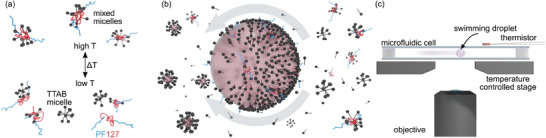
Schematics of the experimental setup and droplet propulsion mechanism. a) With increasing/decreasing temperature, there is an increased/decreased affinity for TTAB to form mixed micelles with PF127. Simplified aggregation schematic.^[^ ^38^ ^]^ b) Droplet propulsion during solubilization: an inhomogeneous distribution of empty TTAB micelles causes a self‐sustaining Marangoni gradient at the oil–water interface. c) Setup: The droplet (diameter d=50±5μm) swims in a quasi‐2D (13mm×8mm×50μm) cell on a temperature controlled microscope stage. All schematics are not to scale (see Figure S2, Supporting Information).

CB15 droplets self‐propel reliably in supramicellar solutions of pure TTAB above 5 wt%.^[^ ^24^, ^39^ ^]^ Briefly put, the swimming is due to oil diffusing from the droplet into TTAB micelles,^[^ ^17^, ^19^, ^22^ ^]^ which removes surfactant from the droplet posterior, while the anterior is replenished by the advection of fresh surfactant (**Figure** **1**b). The key point here is that the empty micelles at the anterior are less thermodynamically stable than the oil‐filled ones at the posterior:^[^ ^22^, ^40^ ^]^ in consequence the critical micelle concentration is higher in front of a moving droplet. The resulting self‐enhancing surface tension gradient drives the droplet forward until it is dissolved. Typically, a CB15 droplet of diameter 50μm will swim in 5 wt% TTAB for 1–2 h. Based on this mechanism, one can define a Péclet number *Pe* of droplet activity that increases with viscosity, droplet radius and surfactant concentration, i.e. chemical activity.^[^ ^18^, ^22^, ^24^, ^25^, ^41^ ^]^ In a surfactant solution where all micelles are oil‐saturated, droplets would not propel, and they are are repelled by gradients in filled and attracted by gradients in empty micelles.^[^ ^39^ ^]^ We may in this sense regard empty TTAB micelles as fresh, and oil‐filled ones as spent fuel.

PF127 is a nonionic triblock copolymer surfactant that in a pure aqueous solution forms micelles with a hydrophobic core of propylene oxide (PPO) and an outer shell of hydrated ethylene oxide (PEO)^[^ ^42^, ^43^, ^44^, ^45^, ^46^, ^47^ ^]^ above the critical micelle temperature, 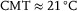 at 4 wt% PF127.^[^ ^44^ ^]^ In the presence of ionic co‐surfactants like TTAB, which bind strongly with PF127, mixed TTAB/PF127 aggregates form (Figure 1a; Section S3, Figures S3–S5, Supporting Information), with excess TTAB forming single‐species micelles.^[^ ^36^, ^38^, ^48^, ^49^ ^]^ It has been found^[^ ^49^ ^]^ that the TTAB binding capacity of PF127 is amplified with increasing temperature, due to an increasing dehydration of the PPO blocks (hydrophobic effect^[^ ^42^, ^50^ ^]^).

Under our experimental conditions, we expect a significant coverage of TTAB at the interface (Figure S6, Supporting Information). Furthermore, CB15 droplets are completely immotile and hardly solubilize in pure PF127 solutions (Figure S7, Supporting Information), while in a mixed TTAB/PF127 medium self‐propelling at speeds ≈20µm/s, comparable to experiments in pure TTAB solutions (cf. Refs. [24, 39] and Figure S8, Supporting Information). We therefore regard TTAB as the primary surfactant mediating the solubilization and the interfacial gradients driving the droplet motion. This activity is controlled by PF127 binding and releasing TTAB micelles in the bulk medium (Figure 1a,b). Thus, the *Pe* of droplet activity *decreases* with *increasing* temperature, as an increasing fraction of TTAB is bound in mixed micelles.

According to literature on the composition of PF127/TTAB aggregates,^[^ ^48^, ^49^, ^51^ ^]^ the amount of bound TTAB in our swimming medium should exceed 1 wt% and further increase with temperature, which is on the order of the amounts required (<5 wt%) to suppress droplet motility,^[^ ^19^, ^24^ ^]^ as observed in the experiments we show below (see also Figure S1, Supporting Information). We further note that the swimming medium is Newtonian, with only weakly temperature dependent viscosity (Figure S11, Supporting Information). The Stokes–Einstein diffusion coefficient for a particle of radius 25μm is $D<1\times 10^{-2}\;\mu m^{2}/s$, such that we can neglect Brownian motion within the duration of observation.

### 2.2 Swimming Dynamics Controlled by Temperature and Fuel Concentration

**Figure 2:**
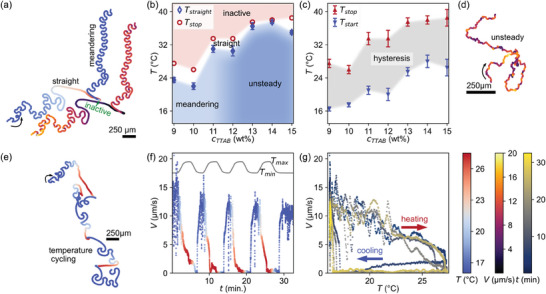
Swimming dynamics controlled reversibly via temperature and fuel concentration. a) One trajectory of a droplet in a mixed surfactant swimming medium, color coded once by droplet speed and once by recorded temperature. The droplet transitions from meandering to straight swimming to arrest during a heating and subsequent cooling ramp with a set rate of 1 K/min. See also Movie S1 (Supporting Information). Color map and scale bars (250μm) apply to all figures in the paper, and concentrations for PF127 and TTAB are always 4 wt% and 10 wt%, respectively, unless stated otherwise. b) Map of the swimming dynamics depending on temperature and surfactant concentration. c) Hysteresis between droplet stop and start transition temperatures. Error bars also apply to *T*_stop_ in (b); experiments done in triplicate with 5–10 droplets each. d) Example of unsteady motion at 15 wt% TTAB and 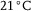 (see also Movie S2, Supporting Information). e) A droplet trajectory during multiple heating/cooling cycles set at 10 K/min and 10 wt% TTAB. See also Movie S3 (Supporting Information). f) Droplet speed versus time for (e), with an inset plot of the recorded temperature ramps. g) Droplet speed versus recorded temperature for (e), showing a hysteresis in the re‐onset of motion during cooling: the arrest during cooling is extended, with a sudden recovery of the initial speed at 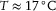.

We begin with an overview of the general swimming dynamics taken from wide‐field video microscopy under changing the ambient temperature, and for a range of TTAB concentrations. The setup contains a quasi‐2D microfluidic cell on a temperature controlled stage (Figure 1c). **Figure** **2**a (Movie S1, Supporting Information) plots a trajectory color‐coded once by speed and once by temperature, recorded at a set heating/cooling rate of 1 K/min, using a swimming medium containing 10 wt% TTAB.

We start at 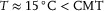. Below 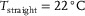, the droplet meanders, i.e., periodically reorients. Above, the motion is straight, gradually slows down and eventually stops at 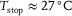. During a subsequent cooling ramp, the droplet remains immotile down to a significantly lower temperature 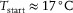, where it abruptly starts to meander again.

As well as to temperature, the swimming dynamics are susceptible to the TTAB concentration. Previous studies on reference systems using a single surfactant species as fuel found a transition from straight to reorienting to unsteady swimming^[^ ^22^ ^]^ with increasing fuel concentration: with activity, the flow speed *U*_*c*_ increases, and therefore *Pe*. Alternatively, similar transitions have been found in experiment if *Pe* was raised via the droplet size or the viscosity of the swimming medium.^[^ ^24^, ^25^, ^41^ ^]^ These dynamics fit into the canonical model for isotropic autophoretic particles,^[^ ^14^, ^18^, ^20^, ^25^, ^52^, ^53^ ^]^ which describes fuel consumption at a spherical interface via a hydrodynamic advection‐diffusion model non‐dimensionalized by *Pe*. The solution ansatz, in the weakly non‐linear limit, decomposes the flow and chemical fields into a series of higher order modes in the interfacial flow and chemical fields, with associated critical *Pe* and growth rates. With increasing *Pe*, starting from the isotropic base, *n* = 0, higher modes like dipolar, *n* = 1, and quadrupolar, *n* = 2 are consecutively excited. At very high *Pe*, numerical studies predict true chaos due to the nonlinearity of the underlying problem.^[^ ^54^, ^55^ ^]^

We have previously investigated the relation between these interfacial modes and straight, meandering and unsteady swimming via dual channel microscopy:^[^ ^24^ ^]^ here, *straight swimming* at barely supercritical *Pe* ∼ 4 corresponds to a pure dipolar interfacial flow structure, *meandering* at intermediate *Pe* ∼ 30 is primarily dipolar with intermittent excitation of a quadrupolar mode that leads to smooth reorientation, while at high *Pe* ≳ 300 the extensile quadrupolar mode dominates, with only short propulsive dipolar intervals, causing a characteristic *unsteady* “stop‐and‐go” motion.

Adding the PF127 co‐surfactant does not appear to change these dynamics at constant low temperature qualitatively, since we find a similar transition from meandering to unsteady motion (Figure 2; Movie S2, Supporting Information) with increasing TTAB concentration. We have summarized these swimming dynamics in a map spanned by temperature and surfactant concentration in Figure 2b. With increasing temperature, we observe a universal transition via straight swimming to eventual arrest. We posit that the temperature dependent TTAB depletion lowers *Pe* below the critical thresholds of higher order interfacial modes, down to *n* = 1 for straight swimming and finally *n* = 0, below the fundamental advection‐diffusion instability. We do not provide a quantitative estimate of *Pe* following,^[^ ^24^ ^]^ as we cannot quantify the temperature dependence of the underlying physical chemistry parameters. Figure 2b also shows a general trend of the transition temperatures to increase with TTAB concentration: for the droplet to arrest, more TTAB needs to be removed from the swimming medium. We found the stop/start hysteresis noted above for all TTAB concentrations in use (Figure 2c).

These state transitions are well reversible with temperature. The experiment shown in Figure 2e–g and Movie S3 (Supporting Information) was recorded at a faster rate of 10 K/min to permit multiple heating and cooling cycles, with dynamics similar to the system cooled at slower rates. Figure 2e,f shows the droplet trajectory color coded by temperature and a corresponding plot of speed over time. The initial motion is recovered after each heating and cooling cycle, apart from a very gradual decrease in maximum speed which we may attribute to droplet shrinkage. The residual motion in the lab frame at peak heating, specifically during the first cycle, appears to be drift in the swimming medium (Figure S13, Supporting Information). We have further analyzed speed versus temperature in Figure 2g, and found a hysteresis cycle with a delayed re‐onset of motion reproducible over multiple heating/cooling ramps. The sample in Movie S3 (Supporting Information) contained three larger droplets (d≈60μm): *Pe* should increase with the droplet radius, and, correspondingly, these droplets switch from reorienting to straight motion at later times and therefore higher temperatures.

### 2.3 Chemical and Flow Fields

We continue with a discussion of the chemical dynamics during the droplet arrest, to motivate the hysteresis in the re‐onset of motion; and of the corresponding flow field to investigate the interfacial mode evolution.

**Figure 3:**
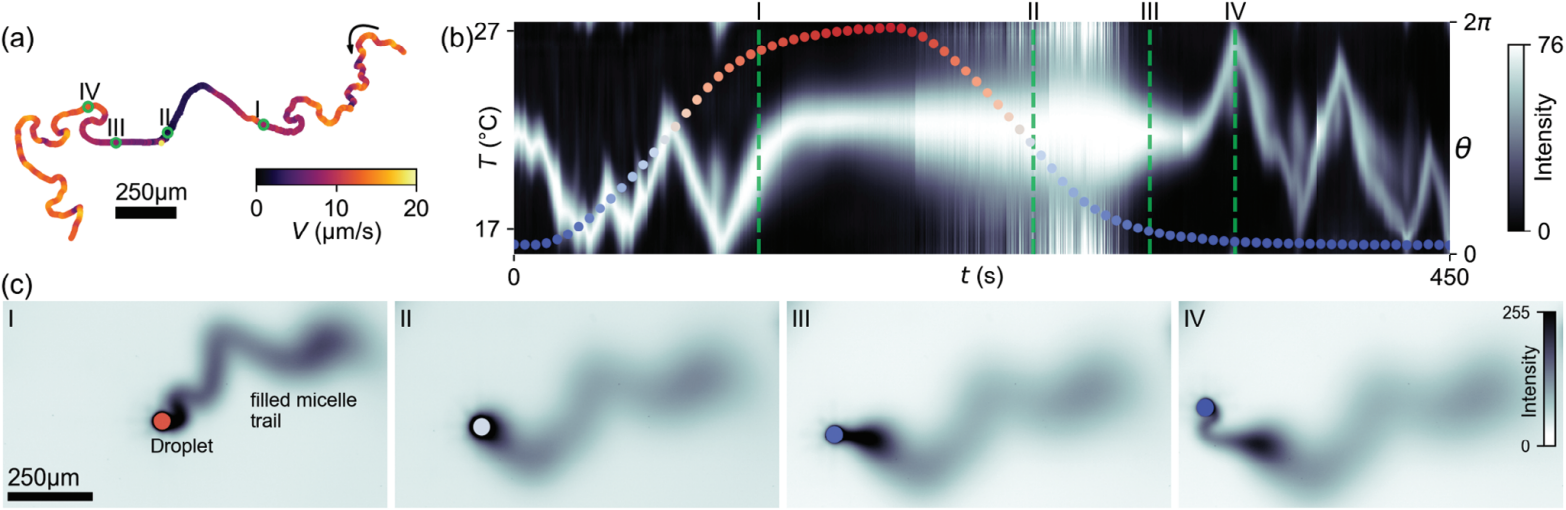
The hysteresis in the re‐onset of droplet motion is caused by spent fuel aggregation. a) Droplet trajectory during heating and subsequent cooling ramp color coded by speed. b) Kymograph showing the evolution of chemical concentration field around the droplet interface and the recorded temperature (colored symbols). c) Snapshots of droplet chemical trails at different temperatures as marked by I–IV in (a) and (b). See also Movie S4 (Supporting Information). 250μm scale bars and color maps as defined in Figure 2.

The self‐generated field of spent fuel in the local environment affects the droplet motility, both via chemorepulsive gradients^[^ ^22^, ^24^ ^]^ and via accumulation of filled micelles, which suppresses the interfacial activity.^[^ ^56^ ^]^ We visualize this field by doping the droplet with the fluorescent dye Nile Red,^[^ ^57^ ^]^ which co‐moves with the oil phase into the filled micelles, and extract the fluorescence intensity *I* from videomicroscopy data (Movie S4). In **Figure** **3**, we analyze these chemical dynamics for one droplet during a heating and cooling cycle, showing a speed‐coded trajectory (a), a kymograph of *I* around the droplet perimeter, θ vs. time and recorded temperature (b), and micrographs at the times marked I–IV (c).

During heating, the droplet transitions from unsteady to straight swimming to immotility (Figure 3a; Movie S4, Supporting Information). (We note again slight drift in the swimming medium, see Section S10, Supporting Information). In the kymograph (Figure 3b), at 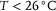, the band corresponding to the chemical trail translates in the angular space due to the reorientation of the droplet (I). At 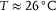, the droplet slows down and comes to a halt. As the system is cooled down to 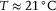, the inactive droplet still solubilizes isotropically, and oil‐filled micelles accumulate around the perimeter θ. Correspondingly, the band in the kymograph widens over the entire angular space (II). We know from experiments in pure TTAB media that these accumulations locally suppress the interfacial activity,^[^ ^53^, ^56^ ^]^ as the density of empty micelle “fuel” is reduced ‐ it follows that in the presence of oil‐filled micelles even more mixed micelles need to disintegrate to restart activity. Thus, the motility transition temperature is lowered, here to 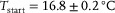, where the droplet escapes the oil‐filled micelle cloud (III) and swims away (IV).

**Figure 4:**
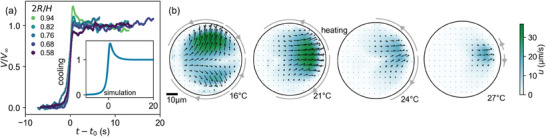
Observation of the transition between passive dissolution and active propulsion. a) Onset of motion during re‐cooling, speed (normalized to steady state *V*_∞_) versus time for multiple runs with *t*_onset_ corresponding to ${\left(dV/dt\right)}_{\max}$. The initial overshoot increases with confinement 2*R*/*H*. Inset: simulation for an isotropic autophoretic particle under comparable conditions (*H* = 2.2*R* confinement, *Pe* = 8), re‐dimensionalized (Section S12 and Movie S6, Supporting Information). b) Internal flow field with increasing temperature, starting at a mixed dipolar/quadrupolar mode (meandering), over a purely dipolar mode (straight) that recedes to the anterior (slowdown). Vectors and color map inside the droplet indicate the velocity field $\vec{u}\left(x,y\right)$; arrows around the perimeter mark the active regions on the droplet interface. Scale bar 10μm.

Before discussing the flow fields, we note two more consequences of oil saturation. First, the hysteresis in Figure 2c is reduced by several degrees if the system is not heated to full droplet arrest, but it is never entirely suppressed (Figure S10, Supporting Information). This can be understood as follows: during the late stage of the heating ramp, the droplet is already dispersing oil into its local environment by recirculation, starting from the posterior ‐ an effect we have also found in self‐throttling pumping droplets in Ref. [56]. During heating, the droplets will come to a stop even before the interfacial activity has fully ceded (see the discussion of **Figure** **4**b below), and self‐propulsion would always need to restart from inside an oil‐rich region as shown in Figure 3c‐II.

Second, the regime of straight swimming appears to be highly localized on cooling (Movies S1 and S6, Supporting Information): the droplet switches after a few seconds to a meandering motion (Movie S1, Supporting Information). We argue here that outside the strongly localized cloud of spent fuel (see Movie S4, Supporting Information; Figure 3c), more empty TTAB micelles have been released, such that the droplet experiences a higher *Pe* once it escapes its self‐generated local trap. We note that during this escape there is a radial gradient from filled to empty micelles, which would also locally rectify the droplet motion.

To analyze the mode evolution causing arrest and sudden onset of motion during heating and cooling (Movie S5, Supporting Information), we added tracer colloids to the oil phase, performed high resolution bright field videomicroscopy and evaluated the internal flow field $\vec{u}\left(x,y\right)$ by particle image velocimetry (PIV) at a series of equilibrated set temperatures. Figure 4b shows the evolution of $\vec{u}$ with increasing temperature. At 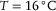, we see a mixed dipolar and quadrupolar flow field (*n* = 1, 2) corresponding to the meandering trajectory in Figure 2a.^[^ ^24^, ^25^ ^]^ At even higher temperature, 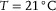, the droplet swims straight, *Pe* decreases and the flow field is purely dipolar (*n* = 1). As the droplet begins to slow down, an inactive region spreads from the droplet posterior (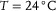). Finally (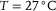), just before the droplet stops (*n* = 0), only a small region at the droplet anterior is active.^[^ ^56^ ^]^ As shown in Figure 3 II, the local environment isotropically saturates with spent fuel while the droplet is immotile.

### 2.4 Simulations

The gradual increase of *Pe* during cooling now allows us to directly observe the fundamental first transition from the immotile base state to self‐propelled motion.^[^ ^14^, ^20^ ^]^ This can be motivated theoretically using hydrodynamic advection‐diffusion models, canonically set out by Michelin et al.^[^ ^20^ ^]^ as follows:

A spherical particle of radius *R* is immersed in a fluid medium containing a chemical fuel at concentration *c*. At negligible Reynolds numbers, the flow is governed by the Stokes equations, $\mu \nabla^{2}\vec{u}=\nabla p$, $\nabla \cdot \vec{u}=0$. The chemical field is coupled by an advection‐diffusion equation,

(1) $$\begin{array}{cccc} \left|\text{Pe}\right|\left(\frac{\partial c}{\partial t}+\vec{u}\cdot \nabla c\right) & =\nabla^{2}c, & \text{Pe} & \equiv \frac{\mathrm{AM}R}{D^{2}}, \end{array}$$

and by the particle consuming fuel at its boundary, $\partial_{t}c\left(R\right)=-A$. The Péclet number is set by the activity A, mobility M and diffusivity *D* of the chemical species and the particle radius *R*. Using a decomposition into squirmer modes and a linear stability analysis around the isotropic base state, *n* = 0, the authors of Ref. [20] find a transition to the propulsive dipolar state, *n* = 1, above a threshold value of *Pe* = 4.

The mixed surfactant approach allows us to observe the growth of the dipolar mode in situ and analyzing it in the context of the canonical model. We performed a simulation of the interfacial instability, following standard protocols,^[^ ^20^, ^58^ ^]^ but here solving for the full 3D problem and adapted to our cell geometry (see Section S12, Supporting Information). We compared our simulation to the experimental droplet speed *V* at the onset of motion, with good agreement between re‐dimensionalized numerical and experimental results (Figure 4a; Movie S6, Supporting Information). For one, the timescales for the onset of motion, of O(1s), and the evolution of the steady state at *V*_∞_, of O(5s), are similar. Second, there is a characteristic overshoot in *V*/*V*_∞_ shortly after the onset of motion, common to various numerical approaches,^[^ ^59^, ^60^ ^]^ and already noted as “surprising.”^[^ ^20^ ^]^ This initial push could be provided by the diffusive cloud of consumed or depleted fuel around the droplet (see e.g., Figure 3c‐II), which causes a radial chemorepulsive gradient. Such gradients would be enhanced in confined geometries:^[^ ^58^, ^61^ ^]^ simulations have found the overshoot to increase with confinement,^[^ ^58^ ^]^ and we find a similar tendency in our experimental data (Figure 4a).

## 3 Conclusion and Outlook

Tuning the dynamics of self‐propelling droplets by temperature‐driven surfactant interactions provides a promising framework to regulate micelle‐mediated^[^ ^62^, ^63^ ^]^ active droplet dynamics: we can now control self‐propulsion from an unsteady or meandering state over quasi‐ballistic propulsion to full arrest without needing to change the chemistry of the system. The gait control is encoded in the swimming medium and does not require complex micro‐engineered swimmer design. Since the hydrophobic effect underlying the temperature dependent complex formation is entropy driven and similar aggregation effects are established for numerous surfactant‐polymer combinations,^[^ ^36^, ^38^, ^48^, ^49^, ^64^ ^]^ this control method likely applies to active droplet models driven by micellar solubilization in general, and could be tested in further studies. For example, autophoretic droplet propulsion is known for SDS surfactant, DEP oil,^[^ ^22^, ^65^ ^]^ and a large number of oil/surfactant combinations tabulated in reviews,^[^ ^12^ ^]^ and one could tune the transition temperatures by using different pluronics like PF88 or PF123.^[^ ^66^ ^]^ The transitions are almost fully reversible, excepting a slight reduction in peak speed that can be attributed to droplet shrinkage.

Our hypothesis—fuel binding by thermosensitive polymer cosurfactants—does not account for the dynamics of adsorbed polymer at the interface, which might also be temperature dependent. However, we argue that these effects are, if present, secondary to the binding and release of TTAB in the swimming medium: generally, the desorption kinetics of large polymers are assumed to be exceedingly slow.^[^ ^50^ ^]^ Thus, if these kinetics were the main drivers of thermoresponsive mode switching, it would not be consistent with our observations of cyclic reversibility and the fast response to changed external conditions, i.e., the instantaneous onset of motion in Figure 4a and particularly the fast, local adaptation to the fuel‐rich medium outside the saturation area.

Our experiments fit into the framework of the canonical theory for autophoretic particles, where the observed dynamic regimes correspond to interfacial modes becoming unstable with increasing or decreasing Péclet number.

While such higher order modes have been documented individually, the fundamental spontaneous transition from an isotropic zero order base state to a first order propulsion state is hard to observe experimentally, as the setup of the experiment usually provides sufficient disturbances to instantaneously set off droplet motion. By a non‐invasive temperature driven crossing of the critical *Pe* threshold, this is now experimentally observable in both chemical and flow signatures. As the idealized theory cited in Equation (1) and its extensions are widely used in numerical work on autophoretic swimmers in complex geometries and confinement, modeling many body interactions, or chaotic dynamics in a huge parameter space, it is important to test its predictive power in an experimental context. The inactive‐active transition analyzed in Figure 4 experimentally matches a common numerical validation case^[^ ^20^, ^55^, ^60^, ^67^, ^68^ ^]^ and highlights the importance of chemical history and spatial confinement in matching numerics and experiment.^[^ ^58^, ^60^, ^69^ ^]^

The approach of depletant‐mediated state control can be combined with a number of methods to guide and functionalize autophoretic swimmers in 2D and 3D^[^ ^31^, ^65^, ^70^ ^]^ Solubilization based swimmers are known to exhibit chemo‐, rheo‐, electro‐, photo‐ and magnetotaxis.^[^ ^28^, ^30^, ^33^, ^39^, ^71^, ^72^, ^73^, ^74^, ^75^, ^76^, ^77^ ^]^ Specifically, photo‐ and magnetotaxis are engineered by a modification of the oil phase like doping, colloidal inclusions or liquid crystalline phases, and therefore independently tunable from the fuel binding in the outer medium.

The co‐surfactant based fuel depletion approach should also apply to solubilization based models that do not activate by spontaneous symmetry breaking of an isotropic base state. These could be ab initio asymmetric Janus droplets, either realized by compound droplets,^[^ ^78^ ^]^ or by adding colloidal patches to the interface,^[^ ^79^ ^]^ which would also be arrested by fuel binding. Moreover, it would be compatible with cargo functionalization ‐ nematic self propelling droplets are stable carriers of configurable aqueous compartments which can be released by dissolution, nematic phase transition or coalescence.^[^ ^80^, ^81^, ^82^ ^]^ We also anticipate that it would inhibit activity in larger droplet‐based autophoretic micropumps.^[^ ^56^ ^]^

Finally, tunable activity can be used to influence aggregation and collective behavior. Autophoretic droplets are known to show complex collective behavior depending on number densities and state of confinement.^[^ ^16^, ^23^, ^57^, ^65^, ^70^, ^71^ ^]^ We note that the cosurfactant mediated arrest mechanism is conceptually different from the self trapping observed previously in single surfactant systems,^[^ ^57^ ^]^ where active droplets were trapped in self generated cages of chemorepulsive trails of oil‐filled micelles, such that the swimmer ensemble is creating its own “chemical landscape.” The latter is a collective phenomenon, the trapping is transient and the *Pe* based state of activity is not modified. This chemorepulsive effect is also observable under PF127 addition: in samples with a higher number density, we have found multiple instances of repulsion interactions (Section S11, Supporting Information). Another collective effect that could be controlled by fuel depletion is the formation of ‘hovercraft’ clusters under gravity in 3D reservoirs:^[^ ^41^, ^65^, ^70^ ^]^ droplets self assemble into crystalline arrangements floating above the container bottom, stabilized by a balance of active upward motion and downward sedimentation. If the activity is suppressed by fuel depletion, these aggregates would sink to the bottom and disintegrate.

In the context of smart, active materials, controllable dynamic states are crucial for programmable motility, function, sensing and adaptation.^[^ ^32^, ^35^ ^]^ In the multi‐scale and multidisciplinary study of synthetic microswimmers presented here, three distinct macroscopic motile states emerge from the nano‐scale control of molecular assembly, and we observe a counter‐intuitive effect of dynamic arrest upon heating. These state transitions are driven by very general thermodynamic principles—our findings therefore offer a general physicochemical design framework for the local and global control of synthetic active matter, beyond the system used here, and offer new avenues to further manipulate different types of active agents from the single to the collective scale.

## Conflict of Interest

The authors declare no conflict of interest.

## Author Contributions

P.R. designed and performed experiments, analyzed data and wrote the paper. P.Rä. and S.M. performed experiments and analyzed data. Y.C. designed and performed simulations. M.J. designed experiments. C.C.M. designed and performed experiments, analyzed data and wrote the paper. All authors proofread the paper.

## Supporting information

Supporting Information

Supplemental Movie S1

Supplemental Movie S2

Supplemental Movie S3

Supplemental Movie S4

Supplemental Movie S5

Supplemental Movie S6

## Data Availability

The data that support the findings of this study are openly available in Zenodo at https://doi.org/10.5281/zenodo.7818660, reference number 13326877.
