# Supplementary material for: Frozen by Heating: Temperature Controlled Dynamic States in Droplet Microswimmers
Source: Adv Mater. 2025 Mar 4;37(15):2416813. doi: 10.1002/adma.202416813 (PMC12004900; doi:10.1002/adma.202416813)
Supplement: Supplementary file 1 — Supporting Information [file ADMA-37-2416813-s003.pdf]

ADVANCED MATERIALS

Supporting Information

for *Adv. Mater.*, DOI 10.1002/adma.202416813

Frozen by Heating: Temperature Controlled Dynamic States in Droplet Microswimmers

*Prashanth Ramesh, Yibo Chen, Petra Räder, Svenja Morsbach, Maziyar Jalaal and Corinna C. Maass**

Frozen by heating: temperature controlled dynamic states in droplet microswimmers. Supporting information

Prashanth Ramesh [1,2] Yibo Chen [2] Petra Räder,[3] Svenja Morsbach [3] Maziyar Jalaal [4] and Corinna C. Maass [1,2,*]

[1] *Max Planck Institute for Dynamics and Self-Organization, Am Faßberg 17, 37077 Göttingen, Germany*
[2] *Physics of Fluids Group, Max Planck Center for Complex Fluid Dynamics and J. M. Burgers Center for Fluid Dynamics, University of Twente, PO Box 217,7500AE Enschede, Netherlands*
[3] *Max Planck Institute for Polymer Research, Ackermannweg 10, 55128 Mainz, Germany*
[4] *University of Amsterdam, Science Park 904, 1098 XH Amsterdam, Netherlands*

(Dated: February 15, 2025)

1. MATERIALS AND CHARACTERIZATION

We obtained CB15 (Synthon Chemicals), TTAB and PF127 (Sigma-Aldrich) and used them as-is. To study the influence of mixed micelles on active droplet motility around room temperature, we varied the TTAB concentration between 9–15 wt%. We note that a low concentration of PF127 requires heating the system to high temperatures to deplete TTAB, causing evaporation issues, while at a higher concentration of PF127 droplets would only be active at low temperatures. This strongly limited the experimentally accessible parameter space for PF127, and we opted to keep the concentration fixed at 4 wt%. An overview of the media compositions for which we observed swimming at room temperature (22 °C) is shown in Fig. S1. Increasing the amount of PF127 suppresses motility, while an excess of TTAB promotes it. The typical molarity ratios for motility are of the order of 1:100.

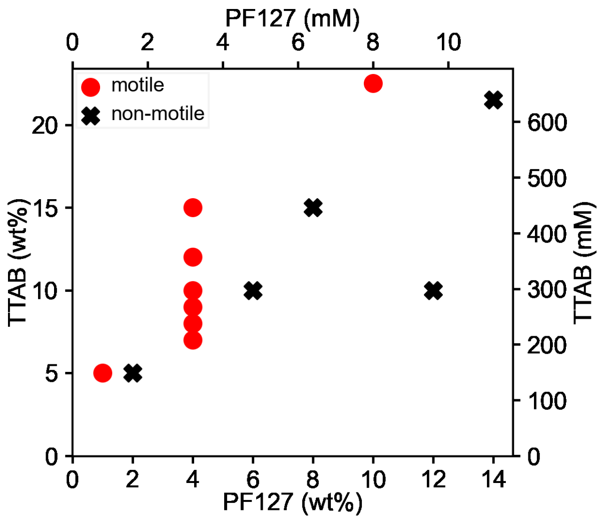

FIG. S1. Motility chart for 50 µm droplets in media of various surfactant composition, taken at room temperature.

2. MICROFLUIDIC EXPERIMENTS

We mass produced CB15 droplets in microfluidic flow focusing devices [24] at a size of (50 ± 5) µm. We fabricated the experimental reservoir, a Hele-Shaw geometry, by spin coating a 50 µm layer of SU-8 photoresist on glass, and creating a rectangular void space of area $(13 \times 8)\,\mathrm{mm}^2$ by UV photolithography. For experiments, we filled it with a dilute droplet emulsion and sealed it with a glass cover slip. The Rayleigh number, estimated for an aqueous medium, a typical length scale of 50 µm and $\Delta T = 10\,\mathrm{K}$, is $Ra \approx 10^{-2}$, ruling out thermal convection effects.

We recorded the motion of active droplets on a brightfield microscope (Olympus IX-81) with a temperature controlled stage (Linkam PE100) which allowed for both heating and cooling protocols. We set the reservoir temperature at the desired initial value for a period of 5 min, after which we started measurements.

We performed temperature ramp experiments with a set heating/cooling rate of 1 K/min, 5 K/min or 10 K/min. As apparent in Fig. 2 (c), the system did not equilibrate instantaneously. We therefore recorded the temperature $T_{\mathrm{meas}} \approx T_{\mathrm{sample}}$ using a thermistor taped to the cover slip sealing the microfluidic reservoir (Fig. 1). The location of the thermistor was separated from the sample volume by a coverslip of thickness ≈ 150 µm, leading to a systematic deviation in the estimated sample temperature T_{sample} due to the gradient between stage, T_{stage}, and room temperature, RT, through the cell (height 1.25 mm, see Fig. S2). Here, for the movies S1 and S3, T_{set} varied between 15 °C and 28 °C, and T_{meas} between (16.2 ± 0.1) °C and (27.3 ± 0.1) °C with RT at 24 °C. Assuming a linear temperature profile via conductive heat transfer, the maximum systematic error would be on the order of 0.2 °C, thus within the experimental error of the transitions documented in Fig. 2.

Movie frames were recorded at 4 frames per second (fps) using a Canon digital camera (EOS 600D) with a digital resolution of 1920 x 1080 px.

We chose heating/cooling rates based on the experimental parameters and the required degree of quantitation. The minimum rate was limited by droplet shrinkage over time. Slower heating rates were feasible for single-cycle experiments, low TTAB concentrations and tem-

* corinna.maass@ds.mpg.de

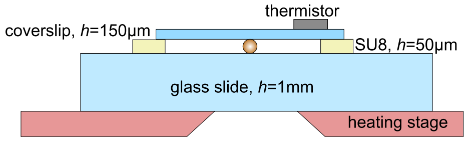

FIG. S2. Cell dimensions: in addition to the heights specified in the figure, the rectangular sample reservoir in the SU8 photoresist layer had an area of 13 mm × 8 mm, and the heating stage had a circular hole for observation of diameter 15 mm.

**Plot**	**Settings**
Fig. 2a	1 K/min
Fig. 2e-g	10 K/min
Fig. 2b,c	5 K/min
Fig. 3	5 K/min
Fig. 4	stepwise heating, ≈ 3 min equilibration before data acquisition

TABLE S1. Summary of heating rates/protocols for the data underlying the figures in the manuscript.

peratures. Table S1 summarizes the protocols for the data underlying Fig. 2-4 and the corresponding supporting videos. For the best determination of $T_{\text{start}}, T_{\text{straight}}$, and T_{stop}, the rate was kept as low as possible, we ensured via the recorded droplet sizes that the polydispersity was low, (50±5) µm, and used small number densities ($< 0.1\,\text{mm}^{-2}$) to exclude mutual trail interactions.

To estimate whether the quasi 2D confinement affects the observed dynamics, we have also recorded the temperature-dependent droplet motion in reservoirs with a larger height of 310 µm and 2 mm, using a swimming medium with 10 wt% TTAB and 4 wt% PF127. We found similar dynamic states and, within the experimental error (see Fig. 2b), the same transition temperatures ($T_{\text{straight}} \approx 23\,°\text{C}$, $T_{\text{stop}} \approx 27\,°\text{C}$) as in the case of the quasi-2D geometry, $h = 50\,\mu\text{m}$ (Fig. 2b).

3. INVESTIGATION OF TTAB/PF127 MICELLE FORMATION

To investigate the formation of individual and mixed micelles in our particular swimming medium, we recorded dynamic light scattering and calorimetry data on solutions of pure and mixed surfactants. The nature of these complexes is according to existing literature highly dependent on concentrations and temperature, however, at the relative concentrations exceeding 1:100 in our system, PF127 micelles are presumably fully broken down for the entire temperature range under investigation [49]. For this reason, we do not expect the CMT of PF127 to be a characteristic quantity in the dynamics of our system.

A. DLS measurements

We performed dynamic light scattering (DLS) measurements for the co-surfactant mixtures used in our experiments on a Malvern Zetasizer Ultra Red. We placed a 1 mL sample in a polystyrene cuvette and recorded the backscattering intensity at 173° between 15 °C and 30 °C. At each temperature, the sample was allowed to equilibrate for 120 s, and all measurements were carried out in triplicate. We have plotted the size distributions for selected temperatures in Fig. S3 and summarized the temperature dependent behavior for all samples via the Z average hydrodynamic diameter, i.e. the "intensity weighted mean hydrodynamic size of the ensemble collection of particles" (Malvern) in Fig. S4. For 4 wt% PF127, there is a significant increase in diameter above 23 °C, corresponding to the formation of PF127 micelles (we note that this appears somewhat higher than the CMT ≈ 21 °C literature value [42, 44]). This increase is suppressed in the presence of TTAB, consistent with the formation of PF127/TTAB complexes seen in literature [38, 48, 49, 64]. We note that, from the Z average, the hydrodynamic diameter of these complexes is somewhat smaller than that of a PF127 monomer, which is in line with existing studies [64]. Further, these complexes appear to be close in size to pure TTAB micelles in a size range < 5 nm near the lower DLS resolution limit, such that the two species probably cannot be resolved in Fig. S3.

B. Differential scanning calorimetry

We investigated the temperature dependent PF127 micellization using differential scanning calorimetry (DSC). For the measurements, we prepared three sample types: (1) PF127 in 4 wt% water, (2) PF127 4 wt% + TTAB 1 wt% in water and (3) PF127 4 wt% + TTAB 10 wt% in water. Solutions were directly transferred into DSC sample aluminum pans (volume 100 µL, Mettler-Toledo GmbH, Gießen, Germany). DSC pans were covered with aluminum lids. DSC measurements were performed on a DSC 823 instrument (Mettler-Toledo GmbH, Gießen, Germany). Heating-cooling cycles were recorded at a heating/cooling rate of 5 K/min between 0 and 80 °C. The measurements were performed under a nitrogen atmosphere with a flow of 30 mL/min. Heating curves were normalized to the sample mass. The endothermic dip in the curve for pure PF127 is consistent with the onset of micellization from the DLS results, and is similarly suppressed under the addition of TTAB.

4. COSURFACTANTS ON OIL-WATER DROPLET INTERFACES

We conducted further experiments to confirm, under our experimental conditions, (A) that TTAB is the dom-

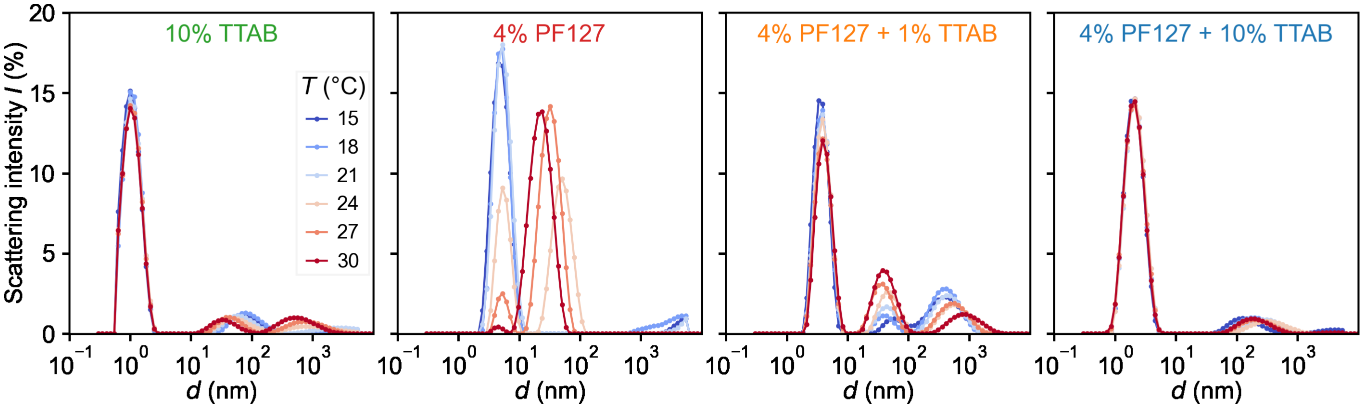

FIG. S3. Dynamic light scattering (DLS) measurements showing the size distributions of four aqueous solutions of PF127 and TTAB with increasing temperature, from backscattering (detector angle 173°). Scattering intensity I vs. hydrodynamic diameter d. Averages over triplicate measurements.

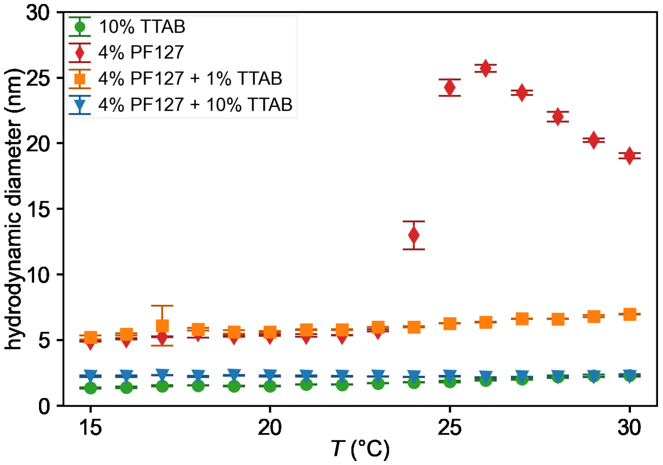

FIG. S4. Intensity weighted Z-average values of the hydrodynamic diameter for each measurement as shown in the DLS series in Fig. S3. The error bars use the standard deviation of the triplicate experimental runs.

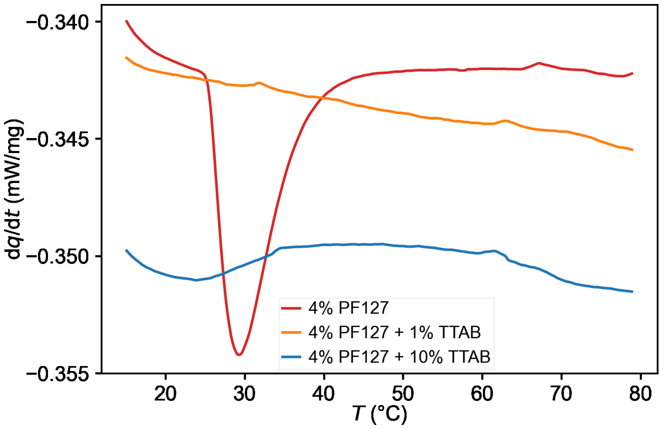

FIG. S5. DSC heating curves of PF127 and TTAB solutions. Measurements were performed with a heat rate of 5 K/min and normalized to sample mass.

inant surfactant at the oil-water interface and (B) that the aqueous solubilization of CB15 is mainly TTAB mediated, as follows.

A. TTAB interfacial coverage based on nematic anchoring

In liquid crystal emulsions, the interfacial anchoring of the nematic director depends on the surfactant in use. Assuming comparable interfacial activity for both substances, we can infer the presence of TTAB at the oil-water interface from investigating the anchoring for CB15's nematic isomer 5CB (at room temperature) under polarized microscopy [83, 84]. We show this in three micrographs in Fig. S6: 5CB droplets (left) in 0.1 wt% TTAB show a cross-shaped interference pattern and a central point defect, consistent with homeotropic (surface normal) anchoring as known for TTAB. On the right, we show a droplet in 0.005 wt% TTAB and 4 wt% PF127, where we observed a bipolar defect pattern, with two opposing defects at the interface (one visible in the micrograph). This pattern is typical for planar anchoring, and we associate it with the large excess of PF127. In the middle image, for 1 wt% TTAB and 4 wt% PF127 (middle), there is only a single point defect, indicating a transition to TTAB mediated homeotropic anchoring already far below the 10 wt% TTAB used in the experiments on motile droplets. For these experiments, we could not use the surfactant conditions in the manuscript, $>$ 5 wt% TTAB and 4 wt% PF127, as in this case the droplets would have been motile at room temperature, distorting the birefringence patterns. However, adding more TTAB would crowd out PF127 at the droplet interface even more, such that we may assume TTAB as the dominant surfactant at the interface under experimental conditions featuring motility.

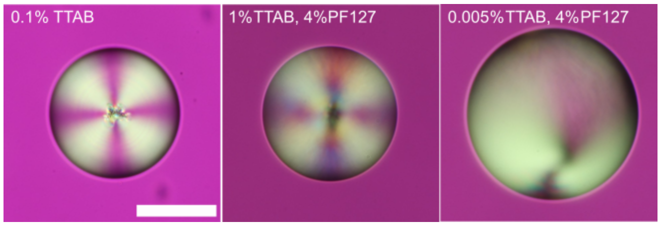

FIG. S6. Polarized micrographs of 5CB droplets in cosurfactant mixtures: left, 0.1 wt% TTAB, shows homeotropic; right, 4 wt% PF127 and 0.005 wt% TTAB, planar anchoring; middle, 4 wt% PF127 and 1 wt% TTAB, primarily homeotropic. Scale bar 50 µm.

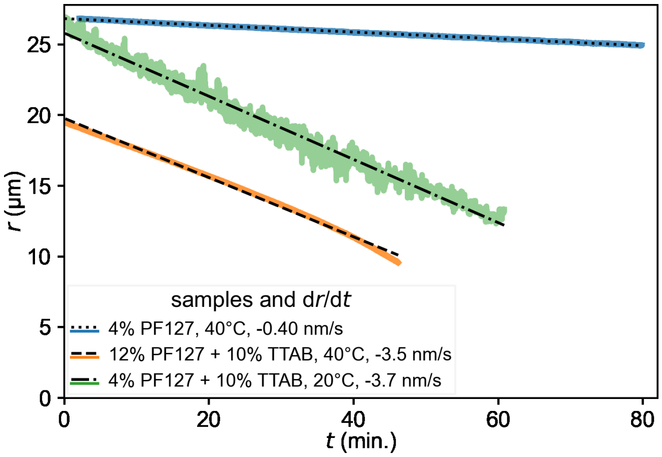

FIG. S7. Decrease in CB15 droplet radius over time for non-motile droplets in 4 wt% PF127 (blue) and 12 wt% PF127 + 10 wt% TTAB (orange) media at $T = 40\,°\mathrm{C}$ (one droplet each at 40x magnification), and for motile droplets in 10 wt% TTAB + 4 wt% PF127 at 20 °C (green, low magnification ensemble average). Dotted lines: linear regression fits to determine the shrinking rates, -0.4 nm/s, -3.5 nm/s and -3.7 nm/s, respectively.

B. Solubilization rate measurements

We measured the shrinking rate of a CB15 droplet for two non-motile cases under bright field microscopy at 63x magnification at an elevated temperature of 40 °C to promote dissolution by enhanced oil diffusion into the aqueous phase, using the concentrations 10 wt% TTAB + 12 wt% PF127 and 4 wt% PF127 (note that pure PF127 at 10 wt% would have formed a hydrogel [46]). Droplet radii and fitted shrinking rates are shown in Fig. S7. We compare these two cases to ensemble averaged data from a lower resolution experiment on motile droplets, in a medium with 10 wt% TTAB + 4 wt% PF127 at 20 °C. Fig. S7 shows the following observations: CB15 does not significantly dissolve in pure PF127 solution (blue). If TTAB is strongly bound by PF127, i.e. at elevated temperatures and elevated PF127 concentration, CB15 droplets will solubilize, but not move (orange). At lower temperatures and with a proportionally higher concentration of TTAB, CB15 will both solubilize and move (green).

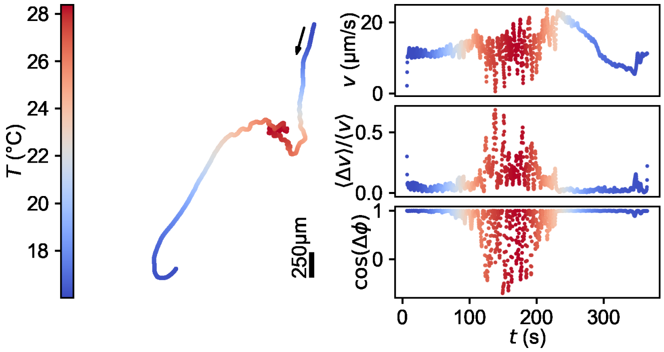

FIG. S8. Temperature dependent droplet dynamics in a solution of pure TTAB at 10 wt%. (a) Droplet trajectory and (b) droplet speed v, and measures of fluctuations in speed, $\langle \Delta v \rangle / \langle v \rangle$, and orientation, $\cos(\Delta\phi)$, over time. With increasing temperature, the droplet motion accelerates and destabilizes. Scale bar 250 µm.

Given that (A) there appears to be a considerable fraction of TTAB at the interface (sec. 4 A) and that (B) droplets neither significantly dissolve or move in pure PF127 (sec. 4 B), we conclude that the droplet motion is primarily driven by TTAB gradients in the oil-water interface, by a mechanism similar to the one driven by micellar solubilization found in pure TTAB media [10, 17, 19, 22]. We also note that the droplet speed is of a similar order of magnitude as the one in pure TTAB ($\approx$ 10–20 µm s^{-1}, Fig. S8), such that any additional adsorbed PF127 at the interface does not appear to impede the mechanism.

5. CONTROL EXPERIMENTS

A. Temperature dependent dynamics in pure TTAB solution

In Fig. S8, we show a temperature-coded trajectory of a droplet studied under the same conditions as the experiments shown in the main manuscript, but in an aqueous solution of TTAB only at 10 wt%. Here, the motion destabilizes with increasing temperature. We illustrate this by temperature-coded plots of three quantities: the speed (top), which increases, but also fluctuates strongly for high temperatures. Further, two simple correlation estimates, both taken over a running time window of $\tau = 2$ s: the standard deviation over average speed, $\langle \Delta v \rangle / \langle v \rangle$ as a measure of unsteadiness in speed, and the cosine of the angle between $\mathbf{v}(t)$ and $\mathbf{v}(t+\tau)$ via their inner product as a measure of rotational fluctuation. Both indicate strong decorrelation at elevated temperatures. We note that this behavior is strongly different from the one in PF127/TTAB mixtures, where speed and unsteadiness decrease with increasing temper-

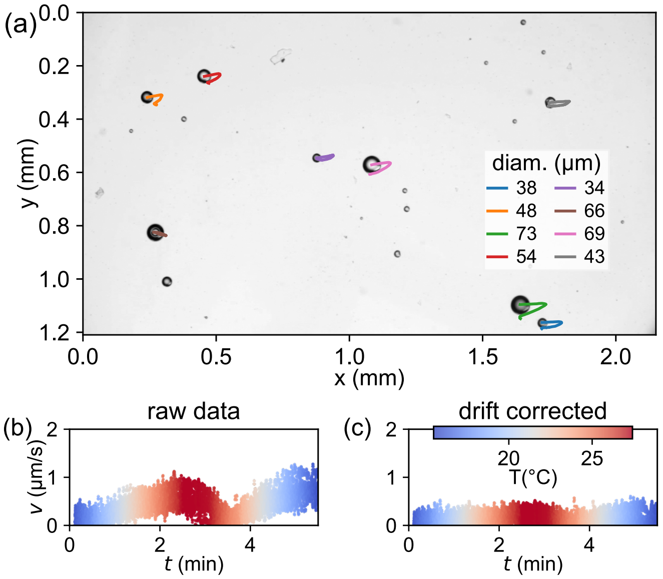

FIG. S9. CB15 droplets are immotile during a temperature ramp between 17 and 28 °C in a 4 wt% PF127 solution. (a) snapshot with trajectories and droplet diameters. (b, c) raw and drift corrected speeds (see Sec. 10).

ature.

B. Temperature dependent dynamics in pure PF127 solution

To further support our hypothesis that the temperature dependent arrest documented in the main manuscript requires interactions between the two cosurfactants, and not found for the surfactants individually, we show in Fig. S9 that there is no active motility for droplets of any relevant size in a 4 wt% aqueous solution of PF127 during a temperature ramp between 17 and 28 °C.

6. REDUCED HYSTERESIS DURING AN INCOMPLETE HEATING RAMP

We have found a reduction in hysteresis to about half the temperature difference when the droplet does not fully stop (i.e., during an incomplete heating ramp, where it slows down to $< 1\,\mu m/s$). Fig. S10 shows an example for 13 wt%, with the speed coded trajectory in (a), speed and temperature over time in (b) and in (c), for comparison, the same quantities for the trajectory plotted in Fig. 2a in the MS. Compared to the $T_{\mathrm{start}}, T_{\mathrm{stop}}$ values for $c_{\mathrm{TTAB}} = 13\,\mathrm{wt}\%$ in Fig. 2c, the hysteresis is reduced almost by half, the droplet doesn't stop entirely and the re-onset of motion is far more gradual than one starting from full arrest (see zoomed in inset in (c)).

7. VISCOSITY MEASUREMENTS

We measured the viscosity of our swimming media on an Anton-Paar MCR 502 rheometer using a cone-plate geometry with a gap width of 0.1 mm. Measurements were carried out at temperatures between 15 °C and 40 °C and shear rates between $0.1\,\mathrm{s}^{-1}$ and $100\,\mathrm{s}^{-1}$ (see Fig. S11). We observe Newtonian rheology and an only weakly temperature dependent viscosity. Thus, while aqueous solutions of PF127 are known to gel at high temperatures, essentially forming a network of micelles, we are still below this non-Newtonian regime at the concentration and temperature range in use.

8. FLOW AND CHEMICAL FIELD MEASUREMENTS

The internal flow field was determined by adding 1 μm diameter tracer Silica particles (Cospheric SiO2MS-1.8) to the oil phase and analysing high magnification videomicroscopy data by particle image velocimetry (PIV). We did not measure external flow fields, since adsorbed PF127 on colloidal tracer particles [85] may cause them to aggregate and impedes accurate PIV measurements.

We recorded videomicroscopy data under a 40x objective using a 4MP camera (FLIR Grasshopper 3, GS3-U3-41C6M-C) at 40 fps at different set temperatures.

To study the sudden onset of motion from an inactive state as the system was cooled, images were recorded at a higher frame rate of 80 fps. Droplet speed over time was calculated from the recorded trajectories.

To visualize the oil-filled micelle chemical trail behind the droplet, we doped the oil phase with Nile Red (Sigma-Aldrich) dye. We performed fluorescent microscopy on an Olympus IX81 microscope with a filter cube (excitation filter ET560/40x, beam splitter 585 LP and emissions filter ET630/75m, all Chroma Technology). Images were captured via a 4x objective using a 4 MP CMOS camera (FLIR Grasshopper 3, GS3-U3-41C6M-C) at 4 fps.

9. DIGITAL IMAGE PROCESSING AND DATA ANALYSIS

We extracted droplet coordinates from bright field microscopy using a sequence of background correction, binarization, blob detection by contour analysis, and minimum enclosing circle fits, and determined trajectories via a nearest-neighbor algorithm using in-house Python scripts building on numpy and opencv. For the strongly overexposed fluorescence data the droplet centroid was calculated via a distance transform algorithm on the fluorescence intensity. The polar intensity map in Fig. 3(b) was derived by taking the intensity in an annular region around the droplet at a distance of 1.2 droplet radii from

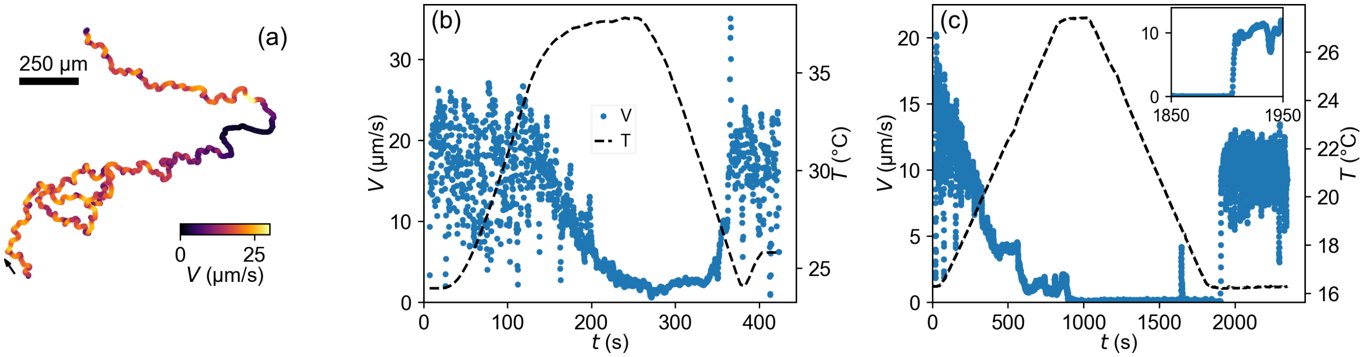

FIG. S10. Reduced hysteresis during an incomplete heating/cooling cycle. (a) speed coded trajectory (b) Speed and temperature versus time (c) The same quantities for a temperature ramp heating to full droplet arrest, using the data from Fig. 1a.

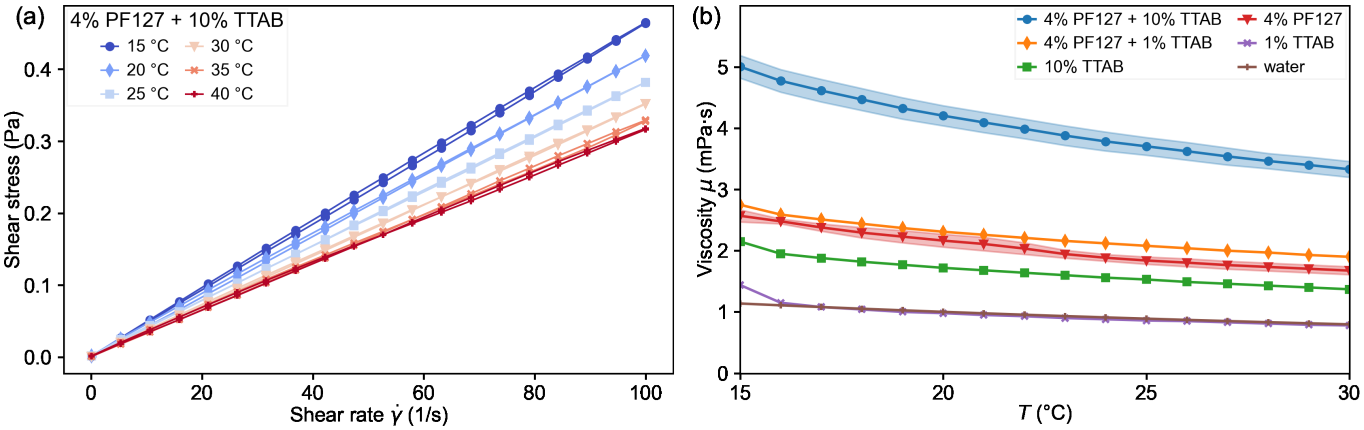

FIG. S11. (a) Shear stress versus shear rates at different temperatures for 4 wt% PF127 + 10 wt% TTAB. (b) Viscosity versus temperature for TTAB and PF127.

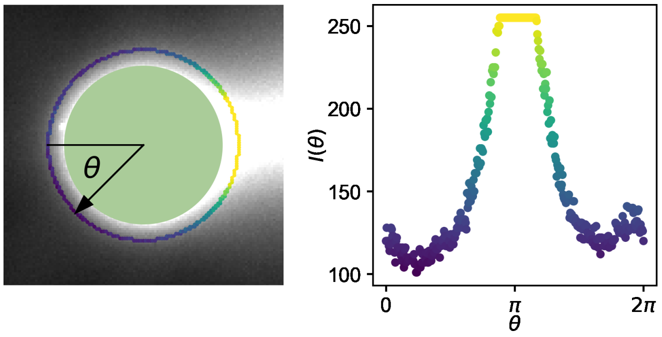

FIG. S12. Protocol to extract fluorescence kymographs from microvideo data, by the example of snapshot III from Fig. 3

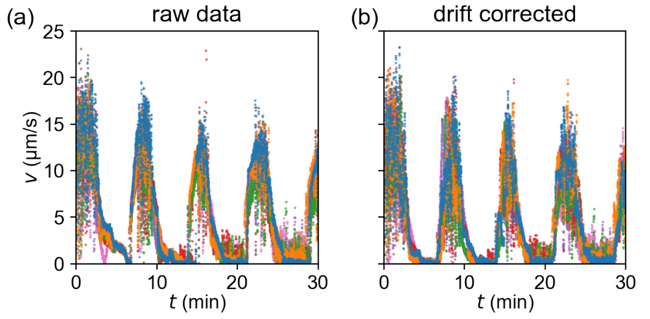

FIG. S13. Drift analysis: Comparison of instantaneous speeds in the ensemble of droplets shown in Movie S3 (see also Fig. 2 e-g), once taken from the raw coordinates (a) and once with the ensemble average position subtracted. During cooling periods, the speed in (b) reverts to zero, indicating that the residual motion in (a) is uniform drift.

the centroid [24, 56, 57], as sketched in supporting figure S12.

Using the time-dependent droplet trajectory and temperature data, we estimated transition temperatures between dynamic states. The error bars for temperature in the regime map (Fig. 2f) represent the maximum variation within three different runs on samples containing on the order of 5–10 droplets each. It should be noted that there is some uncertainty associated with estimating T_{straight} and T_{stop} from low magnification droplet trajectory data. As seen in high magnification data in Movie S5, a droplet could appear to be stationary even though it shows internal activity through the motion of tracer particles. Moreover, the transition between a smooth re-

orientation and straight motion is somewhat gradual.

We performed PIV analyses using the Matlab PIVlab module [86] with a multi-pass interrogation window of 64 x 64 pixel and 32 x 32 pixel with 50% overlap. The spatial resolution of the PIV output was 4.3 µm/px.

10. DRIFT ANALYSIS

Some experiments feature residual motion in the lab frame on the order of ≈ 1–2 µm/s during high temperature states that we identified as arrested and inactive. We believe this is due to drift in the swimming medium, possibly due to the response of the microfluidic cell to the thermal ramp, for the following reasons: (a) In Movie S5, there is no internal flow detectable from the colloidal tracers at high temperature. (b) The fluorescent trails in Movie S4 shift together with the droplets. (c) The residual speed in Fig. 2f is reduced to almost zero if the droplet positions are shifted by the ensemble average, i.e. removing collective translation (Fig. S13). (d) The inactive droplets in Fig. S9 collectively drift to the right on heating and to the left on cooling. Since a correction for the ensemble average similar to Fig. S13 and S9 would add considerable noise during periods of active undirected motility, it is not applied in Fig. 2f. The fluorescence kymographs in Fig. 3 and the PIV data in Fig. 4 take the droplet center as the coordinate origin, and are therefore not susceptible to drift.

11. ADDITIONAL DATA: TRAIL INTERACTIONS

Autophoretic droplets are known to show complex collective behavior depending on number densities and state of confinement. We previously observed transient trapping in pure TTAB solutions [57] at constant $Pe \approx 4$, where active droplets were trapped in self generated cages of chemorepulsive trails, such that the collective is creating its own 'chemical landscape'. Under addition of a cosurfactant, the droplets will still modify their chemical environment in a similar manner. Fig. S14 shows exploratory data taken from an experiment with an increased number density around 4 mm^{-2}, at 4 wt% PF127 and 10 wt% TTAB, with the sample temperature increasing from 17 to 25 °C. Red arrows identify two representative trail collisions, where droplets are repelled by the persistent filled micelle signature of other droplets.

12. NUMERICS

We simulate a diffusiophoretic particle of unit radius initially located at the center of a domain of size $L_x = 10$, $L_y = 100$, $L_z = 2.2$ with 201 × 2001 × 45 grids at subcritical Pe. The particle is propelled by diffusiophoresis, a type of microswimmer similar to active droplet,

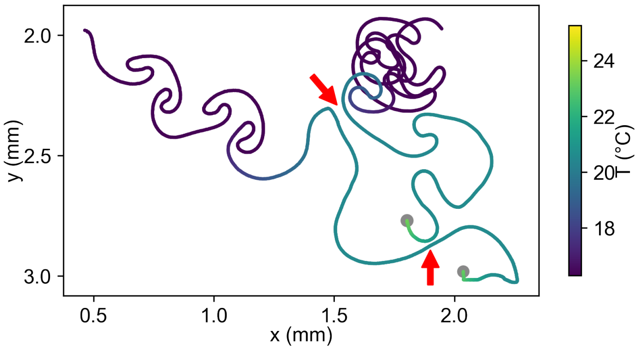

FIG. S14. Collective effects: Similar to droplets swimming in pure TTAB solution, droplets are repelled from each other's trajectory in TTAB/PF127 solutions of 10 and 4 wt%, respectively. Two representative trajectories, color coded by the ambient temperature, with observed collision events marked by arrows. Grey circles represent the measured droplet sizes.

as both move in response to the forces at the surfaces, which depend on the local chemical concentration field (see also [52]). The chemical reaction takes place at the particle surface and whenever there is a chemical concentration difference along the surface, a flow is generated within the interaction layer near the solid surface, with thickness λ of nanometers, which propels the particle forward.

We use the same non-dimensional governing equations and boundary conditions at the particle interface as those in previous studies [20, 67] (here, solving for the full 3D problem as opposed to the axisymmetric analytical approach). The governing equations are given as

$$\frac{\partial c}{\partial t} + \boldsymbol{u} \cdot \nabla c = \frac{1}{Pe} \nabla^2 c, \tag{S1}$$

$$\frac{\partial \boldsymbol{u}}{\partial t} + (\boldsymbol{u} \cdot \nabla)\boldsymbol{u} = -\nabla p + \frac{Sc}{Pe} \nabla^2 \boldsymbol{u}, \quad \boldsymbol{\nabla} \cdot \boldsymbol{u} = 0, \tag{S2}$$

where c is the concentration, u the velocity. Pe is the Péclet number, which is the ratio of advection to diffusion and Sc is the Schmidt number, which is the ratio between the momentum and mass diffusivities:

$$Pe = \frac{M \alpha L}{D^2}, \quad Sc = \frac{\nu}{D}. \tag{S3}$$

where M is the mobility, $M \sim \pm k_B T \lambda^2/(\rho\nu)$ with k_B the Boltzmann constant and T the temperature, ρ the density, ν the viscosity, and D is the diffusion coefficient.

The boundary conditions are given as:

$$\partial_n c = -1 \quad u_s = \nabla_s c, \tag{S4}$$

where $\partial_n c$ represents the concentration gradient at the direction normal to particle surface, u_s the slip velocity and ∇_s is the tangential gradient. The top and bottom boundaries (at z direction) of the domain are set as solid

walls, and all other domain boundaries (at x and y directions) are set as periodic.

We used a central second-order finite difference scheme to spatially discretize the governing equations, with homogeneous staggered grids used in both the horizontal and vertical directions. The equations are integrated by a fractional-step method, with non-linear terms computed explicitly using a low-storage third-order Runge-Kutta scheme and the viscous terms computed implicitly by a Crank-Nicolson scheme [87]. For the particle boundary, we make use of the moving least squares (MLS) based immersed boundary (IB) method, where the particle interface is represented by a triangulated Lagrangian mesh [88]. For the detailed numerical methods and validation, we refer to [67].

In simulations, we observed the onset of symmetry breaking for $Pe \gtrapprox 6$; the velocity profile in the inset of Fig. 4a is taken from a simulation with $Pe = 8$. In Fig. 4(a), the numerical timescale has been redimensionalized using the characteristic time and velocity scales t^* and V^* following [20, 58]

$$Pe = \frac{R \cdot V^*}{D} \qquad t^* = \frac{R}{V^*} = \frac{R^2}{Pe \cdot D}$$

with $D \approx 5 \times 10^{-10}\,\mathrm{m^2/s}$ for the Stokes-Einstein diffusivity of a surfactant monomer [24], $R = 25\,\mu\mathrm{m}$ and $Pe = 8$.

13. SUPPLEMENTARY MOVIE CAPTIONS

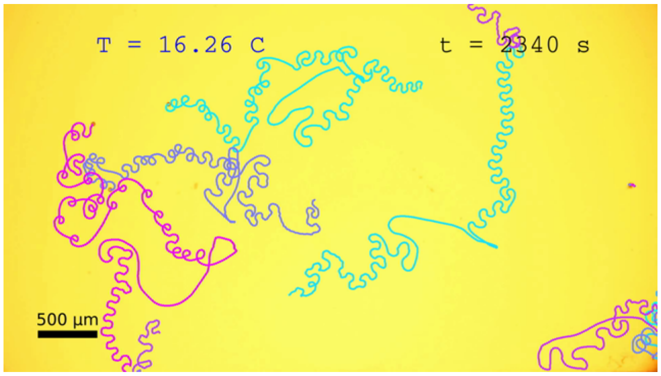

Movie S1. Temperature dependent droplet dynamics at 4 wt% PF127 + 10 wt% TTAB, showing a reversible transition from meandering to straight swimming to arrest during a heating and subsequent cooling ramp with a set rate of 1 K/min.

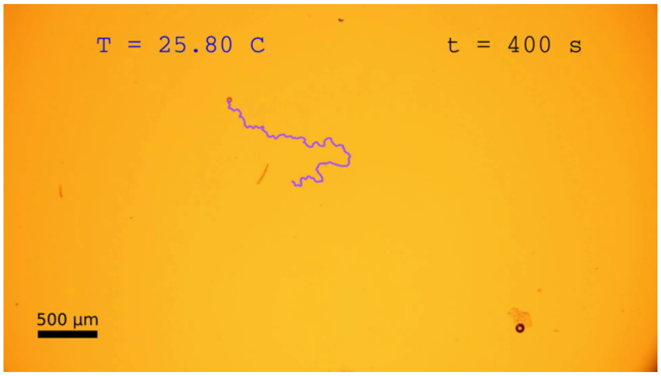

Movie S2. Temperature dependent droplet dynamics at 4 wt% PF127 + 13 wt% TTAB, showing a reversible transition from unsteady to straight swimming to arrest during a heating and subsequent cooling ramp with a set rate of 5 K/min

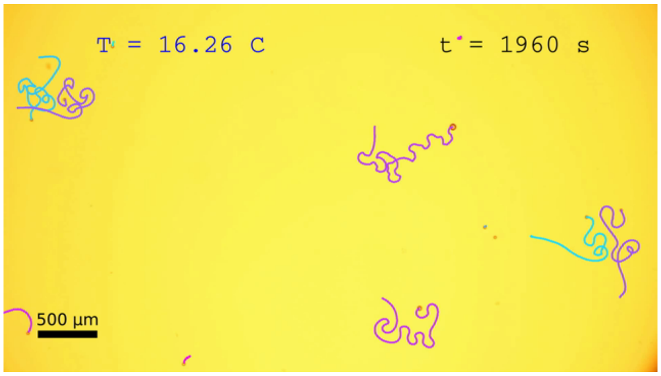

Movie S3. Droplet trajectories at 4 wt% PF127 + 10 wt% TTAB, during multiple heating and subsequent cooling ramps at a set rate of 10 K/min.

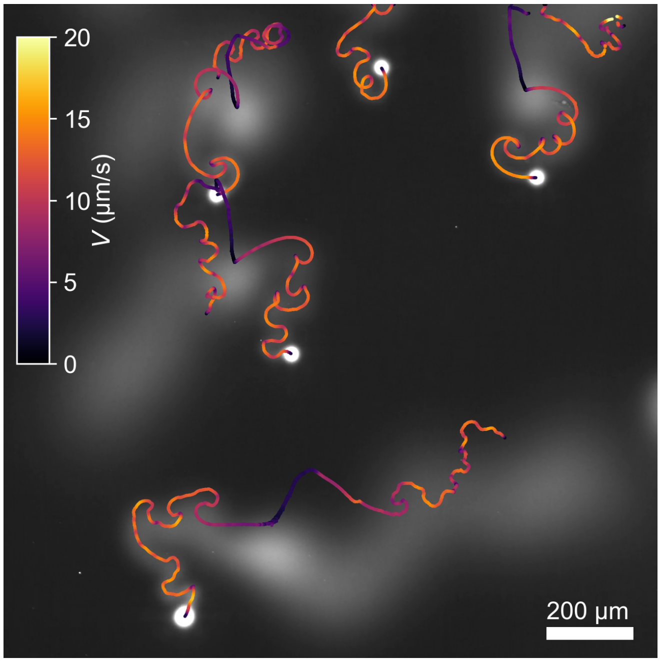

Movie S4. Fluorescent microscopy visualizing droplet chemical trails upon heating and subsequent cooling ramp with a set rate of 5 K/min.

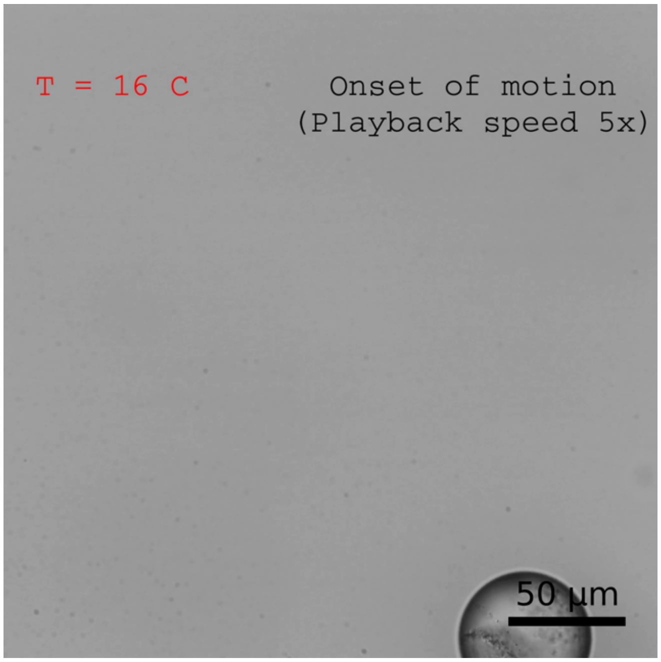

Movie S5. Internal flow visualization with increasing temperature, starting at a mixed dipolar/quadrupolar mode (meandering), over a purely dipolar mode (straight) that recedes to the anterior (slowdown) and onset of motion during cooling. Excerpts recorded during one continuous experiment, playback (at 40 fps) corresponding to real-time during heating, sped up by 5x during cooling.

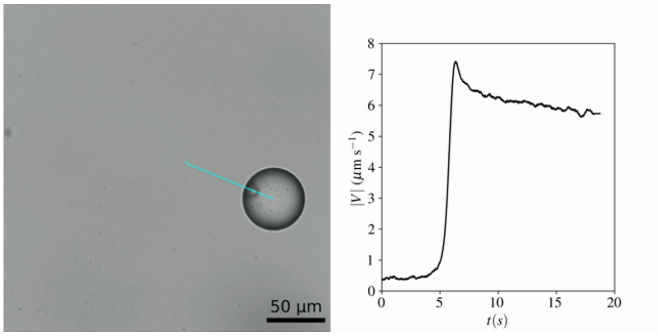

Movie S6. Onset of active motion during re-cooling in a droplet containing tracer colloids, with a superimposed trajectory and the recorded droplet speed V. Experimental duration 18 seconds.
